# *APOE* and *COMT* polymorphisms are complementary biomarkers of status, stability, and transitions in normal aging and early mild cognitive impairment

**DOI:** 10.3389/fnagi.2014.00236

**Published:** 2014-09-05

**Authors:** Roger A. Dixon, Correne A. DeCarlo, Stuart W. S. MacDonald, David Vergote, Jack Jhamandas, David Westaway

**Affiliations:** ^1^Department of Psychology, University of AlbertaEdmonton, AB, Canada; ^2^Neuroscience and Mental Health Institute, University of AlbertaEdmonton, AB, Canada; ^3^Department of Psychology, University of VictoriaVictoria, BC, Canada; ^4^Centre for Prions and Protein Folding Diseases, University of AlbertaEdmonton, AB, Canada; ^5^Department of Medicine (Neurology), University of AlbertaEdmonton, AB, Canada

**Keywords:** *APOE*, *BDNF*, *COMT*, mild cognitive impairment, Victoria Longitudinal Study

## Abstract

**Objective:** Research has reported associations among selected genetic susceptibility biomarkers and risk of (a) normal cognitive aging decrements, (b) established mild cognitive impairment (MCI), and (c) sporadic Alzheimer's disease (AD). In focusing on the transitional normal-to-early MCI phase, we examine associations among three theoretically relevant polymorphisms (*APOE [rs429358, rs7412], BDNF [rs6265], COMT [rs4680]*) and both baseline cognitive status (MCI vs. normal aging) and two-wave (four-year) longitudinal stability or change profiles. The latter included three profiles: (a) stable as normal aging, (b) stable or chronic impairment (MCI-to-MCI), and (c) emergence of impairment (normal-to-MCI).

**Method:** Genotyped older adults (*n* = 237 at baseline; age range = 64–91; 62% women) from the Victoria Longitudinal Study were examined for (a) independent and interactive associations of three genetic polymorphisms with (b) two objectively classified cognitive status groups (not-impaired controls (NIC) and MCI) at (c) both baseline and across a two-wave (four-year) longitudinal interval.

**Results:** First, logistic regression revealed that the presence of at least one *APOE ε4* allele (the risk factor for AD) was linked to greater baseline risk of objective MCI. Second, multinomial logistic regression revealed that (a) the presence of an *APOE ε4* allele was associated with an increased risk of 4-year MCI status stability (chronicity), and (b) the *COMT* homozygous risk genotype (G/G or Val/Val) was associated with an increased risk of both MCI-to-MCI stability (chronicity) and emerging NIC-to-MCI conversion.

**Discussion:** Both chronicity and emergence of objectively classified early cognitive impairment may be genetically heterogeneous phenomena, with influences from a panel of both normal cognitive aging (*COMT*) and AD-related (*APOE*) polymorphisms.

## Introduction

The often lengthy and subtle pathophysiological changes linking normal aging and diagnosable sporadic (late-onset) Alzheimer's disease (AD) are continuous, differentiable, detectable, and worthy of study as a classifiable phase in growing numbers of older adults (e.g., Albert et al., 2011; McKhann et al., 2011; Sperling et al., 2013). Known as mild cognitive impairment (MCI), the key clinical characteristics, markers, and classification procedures have evolved toward a cluster of shared perspectives, related methods, and clinical health implications (e.g., Ritchie et al., 2001; Winblad et al., 2004; Petersen and Knopman, 2006; Albert et al., 2011; Geda and Nedelska, 2012; Lin and Neumann, 2013; Sperling et al., 2013). Briefly, summary principles include: (a) MCI is defined as a mild but clinically relevant neurocognitive dysfunction emerging in a dynamic and variable distribution between normal decline and dementia; (b) biomarkers and risk factors for MCI-related transitions can lead to preclinical targets for earlier interventions to prevent or delay AD; and (c) much work is needed to classify, track, and identify markers of MCI-related transitions (Payton, 2006; Golde et al., 2011; Hurd et al., 2013; Risacher et al., 2013).

Recent research has focused on detecting biological (e.g., genetic risk) and environmental (e.g., lifestyle) factors that discriminate cognitively normal older adults from MCI groups (e.g., Woodard et al., 2012; Anstey et al., 2013). New longitudinal data have revealed evidence of varying stabilities in status, especially in MCI transitions (e.g., Albert et al., 2011; Dolcos et al., 2012; Risacher et al., 2013). This implies that MCI status may be a dynamic, heterogeneous, multi-faceted, and variable phenomenon (Palmer et al., 2002; Koepsell and Monsell, 2012). By definition, MCI involves relatively lower levels of performance or accelerated decline—but not deficits that interfere with everyday functioning or that merit a dementia diagnosis (Albert et al., 2011).

Recommended longitudinal research requires several key classification elements. First, initial status assessment could be confirmed independently at a second session separated by a clinically meaningful interval (e.g., Vandermorris et al., 2011). At baseline, early or transitional MCI classifications may not be etiologically pure, diagnostically firm, or projected to follow an inevitable path to dementia. Second, the phenotypic heterogeneity and multidirectionality of normal cognitive aging (e.g., Dixon et al., 2012; Josefsson et al., 2012) may be reflected also in early transition phases of MCI (Koepsell and Monsell, 2012). Third, multiple factors (e.g., genetic, physiological, health, environmental) contribute to a wide range of normal and early-impaired cognitive aging changes (Fotuhi et al., 2009; Anstey, 2014). Fourth, markers may be independently or interactively (e.g., synergistically) used to discriminate normal aging and early MCI (e.g., Forlenza et al., 2010; Lanni et al., 2012; Risacher et al., 2013). Fifth, classifying individuals in provisional MCI status requires assessments that (a) differentiate normal aging decrements from those prodromal for dementia, (b) produce repeatable (stable) status over longitudinal time, (c) are sensitive to early transitions that emerge during the study period, or (d) are associated with eventual conversion to AD or a related disorder. Finally, MCI classification schemes can be characterized by the extent to which they fulfill these validity-related process and outcome criteria (e.g., Gauthier et al., 2006; Dolcos et al., 2012).

We used objective criteria to identify cognitive deficits vis-à-vis age and education level (e.g., Ritchie et al., 2001; Dixon et al., 2007; de Frias et al., 2009; Plassman et al., 2010; Dolcos et al., 2012). These objective-based procedures coordinate with consensus recommendations but are also transferable across research settings, including clinics, labs, and data archives. For example, using these objective classification procedures we found that select functional biomarkers (e.g., blood pressure, body mass index) could (a) differentiate not-impaired control (NIC) from MCI groups at baseline, (b) establish high levels (70–80%) of status and cognitive stability over a 4-year longitudinal period, (c) provide sensitivity to subtle status transitions (de Frias et al., 2009; Dolcos et al., 2012; Dixon and de Frias, 2014), and (d) relate to underlying neurobiological markers via metabolomics analyses (Zheng et al., 2012). Informing present research on genetic markers of non-demented aging and MCI are studies of both susceptibility genes for sporadic (late-onset) AD (e.g., Bertram et al., 2007) and candidate genetic associations and interactions (e.g., Martinez et al., 2009; Harris and Deary, 2011; Laukka et al., 2013; Sapkota et al., 2014). Examining associations with emerging MCI is a promising research direction (Albert et al., 2011; Brainerd et al., 2011; Izaks et al., 2011; Risacher et al., 2013; Caselli et al., 2014). We now test the role of specific genetic markers of normal cognitive aging and AD, as they relate to early phases of mild cognitive impairment status and change, with the general goal of promoting early detection with its promising intervention possibilities (Reinvang et al., 2010; Risacher et al., 2013).

Both non-demented cognitive aging and sporadic AD are genetically heterogeneous (Harris and Deary, 2011). Early MCI conditions may reflect genetic influences from polymorphisms related to both normal cognitive changes and AD outcomes. We assembled new data from the Victoria Longitudinal Study (VLS) to test whether three commonly associated genetic markers of normal cognitive aging or neurocognitive dysfunction are also associated with objectively classified (a) early MCI status (vs. non-demented aging) at baseline, (b) a 4-year MCI-related status stability profile reflecting chronicity of impaired status, and (c) a 4-year MCI-related status profile reflecting a transitional phase from normal aging to MCI. Three genes commonly implicated in aging and neurocognitive functioning are Apolipoprotein E (*APOE*, rs429358 and rs7412), Brain-derived Neurotrophic Factor (*BDNF*, rs6265), and Catechol-O-Methyl Transferase (*COMT*, rs4680). *APOE* is involved in lipoprotein metabolism. The well-known ε4 variant is the largest known genetic risk factor of sporadic AD and has been implicated in normal cognitive aging decline (e.g., Jorm et al., 2007; Kozauer et al., 2008; Wisdom et al., 2011; Schiepers et al., 2012; Davies et al., 2014). The other two alleles of *APOE* are ε2 and ε3, with (a) the former considered potentially protective and (b) the latter considered neutral for cognitive and neurodegenerative disease. The molecular foundations of *BDNF* and *COMT* associations with neurocognitive performance have been well-described (e.g., Savitz et al., 2006). Briefly, *COMT* codes for an enzyme that degrades catecholamine neurotransmitters such as dopamine in the synaptic cleft. The polymorphism is represented by a valine (Val or G) and methionine (Met or A) substitution, with the latter reducing dopamine degradation. *COMT* Val carriers (both Val-Val and Val-Met) are considered relatively at risk for specific cognitive deficits with aging (e.g., Barnett et al., 2008; Wishart et al., 2011) and possibly MCI (Martinez et al., 2009; Lanni et al., 2012). *BDNF* is a neurotrophic factor involved in neuronal survival and plasticity. *BDNF* expression declines with aging and it is associated with memory performance. The single polymorphism is represented by a Val and Met substitution, with the latter associated with lower hippocampal volume and reduced memory (and cognitive) performance in aging (Miyajima et al., 2008; Tapia-Arancibia et al., 2008), although direct associations are inconsistent (Forlenza et al., 2010; Mandelman and Grigorenko, 2012). For *BDNF*, Met carriers (both A/A and A/G) are at risk. In this study, each of the genes offered potential risk for MCI status classification and MCI status stability over time [i.e., *APOE* risk = ε4 carrier; *COMT* risk = Val carrier (G/G and A/G); *BDNF* risk = Met carrier (A/A and A/G)].

Of the three genetic polymorphisms, *APOE* is the theoretically most likely associate of MCI status and stability (Brainerd et al., 2011; Harris and Deary, 2011). Several recent studies that linked *APOE* ε4 status to relatively late risk of preclinical progression to AD (e.g., Lane et al., 2008; Barabash et al., 2009; Elias-Sonnenschein et al., 2011; Reinvang et al., 2013). Until recently the ε4 variant (including ε4/ε4 and ε4/ε3 combinations) has been inconsistently linked to MCI status, likely reflecting both clinical and methodological differences in status classification (e.g., Brainerd et al., 2011; Risacher et al., 2013). The present study extends a growing collection of results in several ways. First, complementing other studies (e.g., Brainerd et al., 2011; Anstey et al., 2013), we focus on older adults enrolled in a longitudinal study (ages 65–91 at baseline). Second, we examine concurrent and two-wave cognitive status stability relationships. Third, we used a standard and fully objective procedure for MCI classification, which may be useful for detecting early preclinical manifestations of impairment and potentially informative for translational goals. Fourth, in addition to *APOE*, we include two selected genetic polymorphisms, both of which are prominently linked with cognitive phenotypes in normal aging (but rarely with MCI) (Harris and Deary, 2011). However, both *BDNF* and *COMT* have been reported as playing indirect or complementary roles in some studies with implications for MCI and dementia (e.g., Hashimoto et al., 2009; Martinez et al., 2009; Forlenza et al., 2010; Rowe et al., 2010). It is conceivable that these two genetic variants often associated with non-demented cognitive aging phenotypes may be sensitive to early cognitive changes associated with preclinical transition processes. To our knowledge, no previous study has investigated the potential genetic links to MCI of all three genes in the same sample, nor to the longitudinal study of MCI status stability (normal, impairment) or instability (emerging impairment).

We test two research questions. First, we use baseline data to investigate whether *APOE, BDNF*, or *COMT* polymorphisms independently or interactively differentiate between not-impaired cognitively (NIC control group) and MCI adults. Second, using longitudinal data, we test whether *APOE, BDNF*, and *COMT* polymorphisms are independently or interactively associated with stability classification. Specifically, we used the stable normal aging two-wave combination (i.e., NIC-to-NIC) as the comparison for testing genetic influences on two theoretically and clinically viable profiles of cognitive status stability: (a) the conversion or declining status profile (NIC-to-MCI) and (b) the stable and chronic status profile (i.e., MCI-to-MCI).

## Materials and methods

### Participants

This research was conducted under full, active, and continuous human ethics approval from prevailing Institutional Review Boards. Written informed consent was obtained from all participants. Participants were community-dwelling older adults from the Victoria Longitudinal Study (VLS), originally recruited through advertisements in the public media and to community groups. The VLS is an ongoing multi-sample sequential investigation of multiple aspects (e.g., cognitive, neuropsychological, health, sensory, and biological) of human aging. Detailed background information on the design, measures, and procedures is available (e.g., Dixon and de Frias, 2004). For this study, we assembled a 2-wave (mean re-test interval = 4.59 years) longitudinal data set by combining data collected during the same time period across VLS Samples 1 and 2, including only participants with genetic data. Specifically, the current Wave 1 (W1) data were from VLS Sample 1 (Wave 5) and VLS Sample 2 (Wave 3). Similarly, the current Wave 2 (W2) data were from VLS Sample 1 (Wave 6) and VLS Sample 2 (Wave 4). Data were analyzed first using only W1 data (cross-sectional) and then using the full 2-wave longitudinal design. At initial intake, VLS exclusionary criteria are implemented for all samples in order to establish relatively healthy cohorts of older adults, most of which proceed to develop aging-related cognitive and physical health conditions (Dixon and de Frias, 2004). For the present study, exclusionary criteria at baseline (W1) included a history of AD or any dementia, psychiatric disturbance or use of psychiatric medication, MMSE scores less than 24, serious cardiovascular or cerebrovascular disease conditions, uncontrolled hypertension, Type 1 diabetes, or history of serious head injury.

The first research question required the W1 baseline sample. Of the W1 participants (*n* = 237), 136 met the criteria (see below) for the NIC group (Age: *M* = 73.12, *SD* = 5.25; Gender: 64% women; Years of education: *M* = 15.21, *SD* = 2.94) and 101 met the standard criteria for the initial MCI group (Age: *M* = 73.75, *SD* = 5.55; Gender: 59.4% women; Years of education: *M* = 14.52, *SD* = 3.08). The second research question required two-wave longitudinal data. At W2, *n* = 218 participants (of the 237) returned for testing (return rate = 92%). We then excluded *n* = 1 for missing scores on the cognitive status classification measures (*n* = 217). With two baseline status groups there were four possible two-wave status stability classifications. For the longitudinal analysis, we focused on the conceptually and clinically three most pertinent stability groups. First, the stable NIC (NIC-to-NIC: *n* = 101; Age *M* = 73.23, *SD* = 5.28; Gender: 61.4% women; Years of Education *M* = 15.55, *SD* = 2.96) group was our benchmark (non-demented aging) comparison. Second, the corresponding declining NIC (NIC-to-MCI: *n* = 25; Age *M* = 72.64, *SD* = 5.30; Gender: 72% women; Years of Education: *M* = 13.92, *SD* = 2.12) group represented status transition (emerging conversion, early MCI) over two waves. Third, the stable MCI (MCI-to-MCI: *n* = 68; Age: *M* = 73.50, *SD* = 5.40; Gender: 55.9% women; Years of Education: *M* = 14.32, *SD* = 3.00) group represented continuing and chronic MCI status. Regarding the fourth (or “reversion;” Koepsell and Monsell, 2012) group, although previously observed in the literature (Palmer et al., 2002; Brodaty et al., 2013), we had no theory-based prediction for it. Therefore, for this and statistical reasons, we classified the MCI-to-NIC group (*n* = 23; Age *M* = 73.83, *SD* = 5.57; Gender: 65.2% women; Years of Education: *M* = 14.30, *SD* = 3.13) but did not include it in the analyses.

### Cognitive status classification procedure

We used a standard and fully objective (non-clinical and non-subtyped), four-step classification procedure, applying it independently at W1 and W2. The systematic procedure has been used in previous VLS studies and is consistent with other research and consensus reports (e.g., Ritchie et al., 2001; Winblad et al., 2004; Dixon et al., 2007; de Frias et al., 2009; Albert et al., 2011; Dolcos et al., 2012; Dixon and de Frias, 2014). The participants represented the complete subset available for genotyping of a somewhat larger VLS study of MCI (Dolcos et al., 2012). The larger sample was from the same population base and thus was used to optimize normative classification for the present subset (see Dolcos et al., 2012; *n* = 416 at W1; *n* = 301 W1-W2 returnees). The VLS MCI classification procedure emphasized objective assessments of cohort-relative performance on a set of five cognitive reference measures, as evaluated in four successive steps. The first step is to stratify the sample by both age (64–73 and 74–95) and education (0–12 or 13+ years). The second step places each individual into one of four resulting age x education cells. The third step is to calculate mean and distributional characteristics for each of five cognitive reference measures. The five psychometrically sound tasks (e.g., Hultsch et al., 1998) represent the theoretical domains of perceptual speed, inductive reasoning, episodic memory, verbal fluency, and semantic memory. In the fourth step, these means and variability are used for within-sample norms and cognitive status classification. Specifically, we used a moderate criterion whereby participants were classified as MCI if they scored one or more standard deviations (SD) below their own age x education group means on one or more of the five cognitive reference tasks. The one SD criterion was previously established and represented an approach that provided a sensitive degree of differentiation appropriate to the present goal of detecting early signs of cognitive impairment (Ritchie et al., 2001; Dixon et al., 2007; de Frias et al., 2009; Dolcos et al., 2012). See description above and Table 1 for the results of the status classification.

**Table 1:** **Sample demographics at wave 1 by initial cognitive status at wave 1 and cognitive status stability (waves 1–2)**.

**Wave 1**	**NIC**	**MCI**	**Wave 12**	**NIC-NIC**	**NIC-MCI**	**MCI-MCI**
***N***						
W1	136	101		101	25	6S
**AGE**						
W1	73.12 (5.25)	73.75 (5.55)		73.23 (5.28)	72.64 (5.30)	73.50 (5.40)
**GENDER (W)**						
W1	64%	59.4%		61.4%	72%	55.9%
**EDUCATION**						
W1	15.21 (2.94)	14.52 (3.08)		15.55 (2.96)	13.92 (2.12)	14.32 (3.00)
**PERCEPTUAL SPEED**						
W1	50.95 (9.36)	44.15 (10.59)		51.58 (9.89)	49.52 (7.69)	45.19 (10.10)
**INDUCTIVE REASONING**						
W1	12.99 (3.38)	9.31 (4.83)		13.34 (3.45)	12.00 (2.86)	8.74 (4.79)
**EPISODIC MEMORY**						
W1	20.01 (2.94)	15.95 (5.99)		20.18 (3.07)	19.06 (2.37)	15.43 (4.12)
**VERBAL FLUENCY**						
Wl	17.26 (5.64)	12.15 (5.99)		17.98 (5.59)	14.72 (5.00)	11.01 (6.26)
**VOCABULARY**						
W1	46.26 (3.90)	42.14 (5.56)		46.41 (3.78)	45.48 (4.10)	41.32 (5.74)

*W1, Wave 1; W2, Wave 2; Wave 12, Wave 1–Wave 2; N, Sample size; NIC, Not impaired controls; MCI, Mild cognitive impairment; Age and education data presented as Average (Standard Deviation): Cognitive domains represent the 5 cognitive reference measures used for cognitive status classification: For the two-wave groups (Wave 12), the data refer to baseline (W1) values*.

### Cognitive reference measures

The five standard measures from the cognitive reference battery (e.g., Dixon et al., 2007; Dolcos et al., 2012) are widely available and documented with older adults. The psychometric properties and administrative details are documented and acceptable according to conventional standards (e.g., Hultsch et al., 1998). Performance at W1 for each subgroup is shown in Table 1.

#### Perceptual speed

Perceptual processing speed was assessed with the Wechsler Adult Intelligence Scale—Revised Digit Symbol Substitution (DSS) task (Wechsler, 1981). Psychometric characteristics of the DSS are well-established in aging and other populations (e.g., MacDonald et al., 2003). The number of correctly completed items was used as the final outcome.

#### Inductive reasoning

Inductive reasoning was assessed with the Letter Series test (Thurstone, 1962), frequently used with older adults. Participants were presented with 20 strings of letters forming a distinct pattern. The outcome was the total number correct.

#### Episodic memory

The VLS word recall task, consisting of immediate free recall of two lists of 30 English words selected from the total set of six equivalent lists (Dixon et al., 2004), was used. Each list consisted of six words from each of five taxonomic categories (e.g., birds, flowers), typed on a single page in unblocked order. The outcome was the average number of correctly recalled words.

#### Verbal fluency

We used the Controlled Associations test (synonyms) from the Educational Testing Service (ETS) kit of factor-referenced cognitive tests (Ekstrom et al., 1976). The outcome measure was the total number of correct synonyms.

#### Vocabulary

The 54-item recognition, multiple-choice vocabulary test was composed by concatenating three 18-item tests from the ETS kit of factor referenced cognitive tests (Ekstrom et al., 1976). The total number of correct items representing the vocabulary score.

### DNA extraction and genotyping

Following completion of the last testing session, saliva was collected according to standard procedures from Oragene DNA Genotek and stored at room temperature in Oragene® disks until DNA extraction. DNA was manually extracted from 0.8 ml of saliva sample mix using the manufacturer's protocol with adjusted reagent volumes. Briefly, samples were incubated for 2.5 h at 50°C after inversion. Samples were transferred to a centrifuge tube and mixed with Oragene® purifier, incubated on ice for 10 min, then centrifuged at 15,000 g for 5 min to pellet the denatured protein. The supernatant was transferred to a new tube and DNA was precipitated by adding an equal volume of 100% ethanol. The DNA pellet was washed with 70% ethanol, dried, and re-suspended with 10 mM Tris, pH 8.0; 1 mM EDTA buffer. DNA was incubated at 50°C for 1 h with occasional vortexing followed by incubation at 4°C overnight to ensure complete rehydration before quantification using a NanoDrop® ND-1000 Spectrophotometer (Wilmington, DE).

Genotyping was carried out with a Polymerase Chain Reaction (PCR) and Restricted Fragment Length Polymorphism (RFLP) strategy to analyze the allele status for *APOE* (determined by the combination of the Single Nucleotide Polymorphisms (SNPs) rs429358 and rs7412), *BDNF* (rs6265), and *COMT* (rs4680). SNP-containing PCR fragments were amplified in 25 ul of 1X PCR reaction mix containing 25 ng genomic DNA, 12.5 pmol of each specific primer (*APOE* Forward: 5'-GGCACGGCTGTCCAAGGA-3'. *APOE* Reverse: 5′-GCCCCGGCCTGGTACACTGCC-3′; *BDNF* Forward: 5′-AAACATCCGAGGACAAGGTG-3′. *BDNF* Reverse: 5′-AGAAGAGGAGGCTCCAAAGG-3′; *COMT* Forward: 5′-GGGCCTACTGTGGCTACTCA-3′. *COMT* Reverse: 5′-CCCTTTTTCCAGGTCTGACA-3′), 6.25 nmol of each dNTP, 1.25U Taq DNA polymerase (New England Biolabs, NEB), 1.5 mM MgCl2 and 10% dimethylsulfoxide (DMSO). Reactions were set up in 96-well plates using the QIAgility robotic system (QIAgen). Specific amplicons were amplified using a program consisting of: denaturation step at 95°C for 2 min, 40 cycles at 94°C for 30 s, 56°C for 30 s, and 72°C for 1 min, and then a final extension at 72°C for 7 min. RFLP analysis was performed after digestion of the PCR amplicons with restriction enzymes (all from NEB) as follows: (a) *APOE*, for 16 h at 37°C with HhaI, and (b) *BDNF* and *COMT* for 16 h at 37°C with NlaIII. RFLP analysis was then performed on a high resolution DNA screening cartridge on a QIAxcel capillary electrophoresis system (QIAgen) with the protocol OL700. The migration of the restriction fragments on 10 or 15% acrylamide gels for each SNP confirmed the analysis.

### Data analysis

All statistical analyses were performed using SPSS version 17.0 statistical software. For the W1 analysis, we used logistic regression to examine whether *APOE, COMT, and BDNF* polymorphisms were associated with initial cognitive group status (NIC vs. MCI). For the longitudinal analyses, we used multinomial logistic regression to examine whether *APOE, BDNF*, and *COMT* polymorphisms were associated with stability of cognitive functioning (stable NIC-to-NIC, unstable NIC-to-MCI, and stable MCI-to-MCI) across the retest interval. In addition to standard independent candidate gene analyses, we also tested whether genetic risk would be magnified in the context of risk alleles combined across two cognitive-related genes. Because of limited cell sizes and power (in some instances) we followed the procedure of computing the two-way interactions, each of which compared the presence of both risk alleles/genotypes with all other possible genetic combinations for those particular genes. The two-way interactions between the test alleles (*APOE* ε4+, *COMT* G/G, and *BDNF* A/A) were computed, as referenced by the three control alleles (*APOE* ε4−, *COMT* A/A, and *BDNF* G/G). We assessed whether these genetic risk factors in two-way combinations (only) increased the risk of (a) MCI classification at baseline and (b) two-wave conversion to MCI (NIC-to-MCI) or MCI chronicity (MCI-to-MCI). Given our a priori directional hypotheses, we employed one-tailed tests. *Post-hoc* logistic regression power analyses (GPower 3.1) indicate that the present study has (a) sufficient power to detect independent (genotype) effects (baseline single-factor power, *M* = 0.90; longitudinal single-factor power, *M* = 0.80), but (b) lower power to detect two-way (*BDNF, COMT, APOE*) interactions (baseline power, *M* = 0.62; longitudinal power, *M* = 0.42). We reported our interactive analyses cautiously, given few prior relevant reports using these polymorphisms for MCI.

## Results

We first report the initial allelic frequency results. For the two research questions age and education were entered into all analyses as covariates. Analyses without education as a covariate (Luciano et al., 2010) showed identical patterns.

### Genotype and allelic frequencies

Consistent with literature indicating that *APOE, BDNF*, and *COMT* polymorphisms may be associated with lower levels of cognitive functioning in older adults (Bruder et al., 2005; Payton, 2009; Cathomas et al., 2010; Wisdom et al., 2011; Laukka et al., 2013), we informally expected that these genetic polymorphisms would show differential frequencies between the two cognitive status groups at baseline and among the four cognitive status stability groups (Brainerd et al., 2011). As can be seen in Table 1, participant demographics did not differ appreciably between waves or groups. Genotype and allele frequencies at baseline and at follow-up are reported in Table 2 (W1) and Table 3 (W1-W2) for each cognitive status and stability group. As seen in these tables, the distributions are reasonable and generally expected. For all three polymorphisms the W1 sample distributions were in Hardy-Weinberg equilibrium: (a) *COMT* (χ^2^ = 0.06, n.s.), (b) *BDNF* (χ^2^ = 0.09, n.s.), and (c) *APOE* (calculated in three groups rendered in terms of presence/absence of the ε4 allele) (χ^2^ = 0.02, n.s.).

**Table 2:** **Frequency of *APOE, COMT* and *BDNF* genotypes and alleles at wave 1, as stratified by cognitive status**.

**Variable**	**NIC**	**MCI**
N	136	101
***APOE***		
No ε4	106 (77.9)	69 (68.3)
≥ 1 ε4	30 (22.1)	32 (31.7)
***COMT***		
A/A	31 (22.8)	20 (19.8)
A/G	80 (58.8)	53 (52.5)
G/G	25 (18.4)	28 (27.7)
***BDNF***		
A/A	8 (5.9)	4 (4)
A/G	36 (26.5)	34 (33.7)
G/G	92 (67.6)	63 (62.4)

*APOE, Apolipoprotein E; ε4, APOE epsilon 4 allele; BDNF, Brain derived neurotrophic factor; COMT, Catechol O-methyltransferase; G, Guanine, Valine amino acid; A, Adenine, Methionine amino acid; N, Sample size; NIC, Not Impaired Controls; MCI, Mild Cognitive Impairment; ≥1 ε4, the presence of at least 1 APOE ε4 allele; data presented as Frequency (Percentage)*.

**Table 3:** **Frequency of *APOE, COMT* and *BDNF* genotypes and alleles across the W1-W2 retest interval, as stratified by cognitive status stability group**.

**Variable**	**Wave**	**NIC-NIC**	**NIC-MCI**	**MCI-MCI**
***N***				
	W2	101	25	68
***APOE***				
no ε4	W2	80 (79.2)	18 (72)	46 (67.6)
≥1 ε4	W2	21 (20.8)	7 (28)	22 (32.4)
***COMT***				
A/A	W2	27 (26.7)	4 (16)	14 (20.6)
A/G	W2	59 (58.4)	13 (52)	35 (51.5)
G/G	W2	15 (14.9)	8 (32)	19 (27.9)
***BDNF***				
A/A	W2	5 (5)	2 (8)	3 (4.4)
A/G	W2	29 (28.7)	4 (16)	20 (29.4)
G/G	W2	67 (66.3)	19 (76)	45 (66.2)

*APOE, Apolipoprotein E; ε4, APOE epsilon 4 allele; BDNF, Brain Derived Neurotrophic Factor; COMT, Catechol O-methyltransferase; G, Guanine, Valine amino acid; A, Adenine, Methionine amino acid; W1, Wave 1; W2, Wave 2; N, Sample size; NIC, Not Impaired Controls; MCI, Mild Cognitive Impairment; ≥1 ε4, the presence of at least 1 APOE ε4 allele; data presented as Frequency (Percentage)*.

### Genetic markers of baseline cognitive status

We used binary logistic regression to examine baseline group differences in (a) cognitive status (NIC vs. MCI) and (b) *APOE, COMT* and *BDNF* genotype. As reported in Table 4, the presence of at least one *APOE ε4* allele was associated with a 1.65-fold higher likelihood of MCI classification at baseline compared to NIC [*p* = 0.047 (1-tailed)]. The *COMT* A/G (Met/Val) or *COMT* G/G (homozygous Val) genotypes were not associated with an increased risk of MCI classification at baseline compared to the *COMT* A/A (homozygous Met) genotype. Similarly, the *BDNF* A/G or *BDNF* A/A (homozygous Met) genotypes were not associated with an increased risk of MCI classification at baseline compared to the *BDNF* G/G (homozygous Val) genotype. The 2-way interactions between *APOE, COMT*, and *BDNF* revealed no significant increased risk of MCI classification at baseline for the interaction terms.

**Table 4:** **Statistical results relating genetic risk factors to cognitive status and stability for *APOE, COMT* and *BDNF***.

**Predictor**	**Wave**	**Status/Stability**	**Genotype**	**P**	**OR**	**95% CI**	**P**
*APOE*	**W1**	**MCI**	**≥1 ε4**	**0.503**	**1.653**	**0.918–2.976**	**0.047**
	W12	NIC-MCI	≥1 ε4	0.383	1.467^*^	0.533–4.038	0.458
	**W12**	**MCI-MCI**	**≥1 ε4**	**0.598**	1.819^*^	**0.892–3.710**	**0.050**
*COMT*	W1	MCI	A/G	0.036	1.037	0.534–2.013	0.458
	W1	MCI	G/G	0.560	1.750	0.798–3.840	0.082
	W12	NIC-MCI	A/G	0.434	1.543	0.455–5.235	0.243
	**W12**	**NIC-MCI**	**G/G**	**1.371**	**3.939**	**0.976**–**15.900**	**0.027**
	W12	MCI-MCI	A/G	0.160	1.173	0.540–2.551	0.344
	**W12**	**MCI-MCI**	**G/G**	**0.956**	**2.601**	**0.998**–**6.775**	**0.025**
*BDNF*	W1	MCI	A/G	0.328	1.388	0.783–2.461	0.131
	W1	MCI	A/A	−0.164	0.848	0.239–3.014	0.400
	W12	NIC-MCI	A/G	−0.673	0.510	0.157–1.661	0.132
	W12	NIC-MCI	A/A	0.278	1.321	0.220–7.930	0.381
	W12	MCI-MCI	A/G	0.048	1.041	0.523–2.102	0.447
	W12	MCI-MCI	A/A	0.782	1.018	0.220–4.718	0.491

*APOE, Apolipoprotein E; ε4, APOE epsilon 4 allele; BDNF, Brain derived neurotrophic factor; β, co-efficient: COMT, Catechol O-methyltransferase; A, Adenosine nucleotide, Methionine amino acid; G, Guanine nucleotide, Valine amino acid; N, sample size; NIC, Not Impaired Controls; MCI, Mild Cognitive Impairment; OR, odds ratio: W1, Wave 1; W12, Wave 1–Wave 2; ≥1 ε4, the presence of at least 1 APOE s4 allele; for APOE analyses, the presence of ≥1 ε4 was compared to the absence of ε4 (reference group): for BDNF analyses, BDNF A/G and A/A were compared to BDNF G/G (reference group) separately; for COMT analyses, COMT G/G and A/G were compared to COMT A/A (reference group) separately; the NIC group was the reference group for W1, whereas the stable NIC group was the reference group for W1-W2 analyses; p values are all presented as one-tailed; stars indicate dose response findings*.

### Genetic markers of two-wave stability of cognitive status

The association between *APOE, COMT*, and *BDNF* genotype with cognitive status stability across the W1-W2 retest interval was examined using multinomial logistic regression. Consistent with previous reports which indicate that *APOE, BDNF*, and *COMT* polymorphisms may be implicated in cognitive decline (Bruder et al., 2005; Blom et al., 2009; Hashimoto et al., 2009; Schiepers et al., 2012), we expected that *APOE, BDNF*, and *COMT* polymorphisms would be associated with NIC instability (i.e., emerging impairment, NIC-to-MCI) and MCI stability (i.e., impairment chronicity, MCI-to-MCI) across the retest interval. As reported in Table 4, the presence of at least one *APOE ε4* allele was associated with a 1.82-fold higher likelihood of MCI-to-MCI stability (chronicity) over time compared to a stable NIC-to-NIC classification (*p* = 0.050, 1-tailed). Although *APOE* ε4 was not associated with NIC-to-MCI decline over time, a dose-response effect for the ε4 allele was noted between the 2-wave cognitive stability groups (i.e., NIC-to-NIC, MCI-to-MCI; Table 4). Although the *COMT* G/G (homozygous Val) genotype was not associated with baseline status, it was associated with both a 3.94-fold higher likelihood of NIC-to-MCI decline and a 2.60-fold higher likelihood of MCI-to-MCI stability (chronicity) over time, as compared to the stable NIC-to-NIC classification group (*p* = 0.027 and 0.025, respectively, 1-tailed). *COMT* A/G was not associated with either two-wave profile. The *BDNF* A/A (homozygous Met) or A/G genotypes were not associated with NIC-to-MCI conversion or with MCI-to-MCI stability over time. The 2-way interactions between *APOE, BDNF*, and *COMT* did not confer any significant associations with the stability and status groups.

## Discussion

We examined risk of objectively assessed MCI (distinguished from non-demented aging) as associated with risk alleles of three relevant polymorphisms. Group membership in three MCI profiles was examined: (a) baseline (W1), with NIC compared with MCI, (b) two-wave stability of MCI status, reflecting chronic and stable condition (i.e., MCI-to-MCI), and (c) two-wave decline in NIC status, reflecting early and emerging conversion to classifiable impairment (i.e., NIC-to-MCI). This study both replicates and extends recent single-wave MCI assessments by including (a) two-wave independent follow-up classifications, (b) tests of the typical *APOE* genetic variant plus two additional variants, and (c) assessments of two-wave patterns reflecting impaired chronicity and emerging conversion.

We discuss the results as organized by the three genetic polymorphisms. First, *APOE* was expected to be a significant marker. The ε4 variant is the best known genetic risk factor for sporadic AD (Albert et al., 2011; Harris and Deary, 2011) and has been implicated in candidate gene studies for normal cognitive decrements (e.g., Small et al., 2004; Kozauer et al., 2008; Liu et al., 2010; Izaks et al., 2011; Wisdom et al., 2011; Davies et al., 2014) and mild cognitive impairment (Brainerd et al., 2011; Reinvang et al., 2013). Brainerd and colleagues reported that the ε4 variant was more represented in older adults classified as MCI than in a non-demented comparison group. Our results are consistent with (and extend) these findings. Using fully objective classification procedures, we observed that the presence of at least one *APOE* ε4 allele conferred increased risk of MCI classification (a) at baseline and (b) for stability (chronicity) over the two-wave interval. We interpret the latter as evidence both for the stability of our MCI classifications and for their usefulness for detecting preclinical decrements early in cognitive aging. Notably, we use the objective, multi-dimensional cognitive reference battery approach to capture mild disorder that (a) may be transient or variable among some such early cases of impairment (Koepsell and Monsell, 2012), (b) may even be reflected by modest deficits (i.e., 1 *SD*) in a single fundamental domain (Dolcos et al., 2012), and (c) do not reflect the potential that differential domain deficits may be indicative across waves and subtypes (Brodaty et al., 2013). For this reason, the classification battery represents five complementary domains of basic cognitive resources. Within the objective VLS classification approach, this is the first evidence regarding genetic associations, but it has previously pointed to MCI associations with executive functioning (de Frias et al., 2009), memory (Dixon and de Frias, 2014), neurocognitive inconsistency (Dixon et al., 2007), functional biomarkers (Dolcos et al., 2012), and molecular-level metabolomics markers (Zheng et al., 2012). Future progress will be achieved when researchers are able to assemble and analyze multifaceted sets of markers relevant to early MCI transitions, stability, and outcomes (Fotuhi et al., 2009; Anstey, 2014). Although we present two-wave data, we must await future waves to discover the extent to which MCI-to-MCI chronicity is also probabilistically linked to eventual AD. For now, the predictions of surrogate outcomes (MCI baseline status and 4-year stability or chronicity) are promising results.

Second, the *COMT* G variant (including Val-Val and Val-Met) has been identified as a risk factor for cognitive deficits with normal aging, given the association with the degradation of catecholamine neurotransmitters (such as dopamine) (Savitz et al., 2006). However, previous results of candidate gene studies have revealed promising but inconsistent association patterns (Barnett et al., 2008; Wishart et al., 2011; Sapkota et al., 2014) and MCI status (Martinez et al., 2009; Yarnell et al., 2014). Had we only examined concurrent data we would have reported no MCI-related associations. Instead, the *COMT* variant emerged as a significant marker of MCI chronicity and transition in the two-wave data. This may be due to its sensitivity to early cognitive aging disorders in performances represented by the present broad-based neurocognitive reference battery, especially among older adults who are transitioning (NIC-to-MCI) or chronically MCI. These dynamic groups may present more homogeneous MCI-related phenotypes than the larger baseline source group. The extent to which the *COMT* G variant (homozygous G/G) was involved in predicting status chronicity and conversion was novel and promising. Together with recent work showing promising synergistic effects (Martinez et al., 2009; Lanni et al., 2012), *COMT* may deserve additional attention in MCI, especially in the context of *APOE*, longitudinal follow-ups, and phenotypes reflecting its neurobiological influence. Perhaps with objective but sensitive assessment procedures, *COMT* may prove to be a candidate genetic susceptibility marker of early cognitive changes associated with neurobiological disruptions that may be secondary or preliminary to AD-related neuropathology. At present, the absence of *COMT* from the pantheon of MCI-related genes may be associated with the fact that, by the time individuals convert to dementia, the genetic associations are overwhelmed by *APOE* and its direct connection to amyloid beta deposition. Future research can investigate whether the apparent separate contributions of *APOE* and *COMT* to MCI status and change continue to be complementary or whether a gene-related would produce synergistic vulnerability (Lindenberger et al., 2008; McFall et al., 2014).

Third, the other polymorphism (*BDNF*) was not statistically associated with the targeted outcomes. In general, *BDNF*-cognition associations are inconsistently observed and difficult to interpret (Mandelman and Grigorenko, 2012). In addition, *BDNF* associations are rarely tested as predictors of clinical or cognitive status or neurodegenerative changes (cf. Forlenza et al., 2010). The mechanisms through which *BDNF* may affect neurocognitive performance are proposed (Savitz et al., 2006; Harris and Deary, 2011) but their relevance to non-normal cognitive status has not been firmly established. As an MCI predictor, *BDNF* may be less relevant for early classification (as in this study) than for later cognitive impairment (Forlenza et al., 2010). We included *BDNF* for two reasons: (a) it could have been related to objective cognitive status as we assess it (via performance on a reference battery including memory and speed markers) and (b) it could have appeared as an associate of cognitive status in the role of interacting influence.

Overall, the findings of this study reveal several interesting independent and complementary genetic associations with non-demented aging and MCI status, including initial, transitioning, and chronic profiles. However, several limitations should be acknowledged. First, not all VLS data were available for the present study, as the first phase of the genetics initiative occurred only recently, thus limiting the overall and (especially) clinical group sample sizes. Further genotyping and follow-up waves are planned. Second, our MCI classification procedures are derived from consensus statements (e.g., Albert et al., 2011) and standardized procedures (e.g., Dixon et al., 2007). They do not include several characteristics sometimes observed in the literature: (a) clinical judgment or personal evaluations, (b) differentiation into subtypes of MCI, or (c) criteria emphasizing established or severe pre-dementia cases (e.g., Winblad et al., 2004; Petersen and Knopman, 2006; Dolcos et al., 2012). Instead, we emphasized (a) replicable and transferable classification (i.e., objective procedures may be translated into multiple research and clinical settings), (b) overall MCI classification based on deficits in multiple possible basic domains as based on the assumption that early or emergent cases may not (yet) be consolidated into subtypes, and (c) use of moderate criteria (i.e., 1 *SD* below the appropriate group mean) so that early cases may be identified, at the risk of some false positives. In the VLS, transitions into MCI and dementia are slowly emerging as the VLS itself “ages.” Emergence of differential subtypes, etiology, and severities will be followed, as they could modify future interpretations. Third, we focus on predictors of emergence, stability, and early transitions in MCI. Extending research to predictors of later transitions (e.g., MCI-dementia) will require larger samples of well-diagnosed dementia patients. Other researchers are also embracing the challenge of investigating characteristics and markers of early MCI (e.g., Kryscio et al., 2006; Anstey et al., 2013; Risacher et al., 2013) using status change or stability as surrogate outcomes.

Fourth, the sample size of the present study was limited because the design required participation in two longitudinal waves. Relevant published studies vary in sample size, age ranges, and availability of longitudinal intervals (Caselli et al., 2007, 2014; Martinez et al., 2009; Forlenza et al., 2010; Brodaty et al., 2013; Risacher et al., 2013). We reported a limitation in the power to detect gene x gene interactions. However, the fact that we did not observe significant synergistic interactions may also be related to other fundamental aspects of the emergence and progression of relatively early MCI in aging: (a) these processes may not have been sufficiently advanced to “recruit” or “require” such interactional contributions, (b) these genetic variants may not be those that are involved in interactional predictions of MCI, and (c) more diverse, more select, more severe, or etiologically purer samples might extend the present results. Much larger studies may be better powered for interaction analyses, but this can balanced if they lack the nuanced or valid cognitive measures or have suboptimal intervals. Therefore, we do not rule out the potential role of gene x gene interactions in predicting the phenomena we address in this study. Fifth, there are a number of unmeasured factors in any study of normal cognitive aging or emergent and longitudinal MCI phenomena (e.g., Fotuhi et al., 2009; Albert et al., 2011; Woodard et al., 2012). One possible avenue for future research includes investigation of multiple AD-related markers (Risacher et al., 2013) or gene x environment (e.g., health; Anstey, 2014; McFall et al., 2014) effects. Sixth, we studied only three genetic variants, and others could be investigated in the prediction of MCI status, stability, and transitions. However, many candidate gene studies of MCI include only one variant (typically *APOE*). We recommend the careful selection of additional polymorphisms based on known underlying neurobiological mechanisms related to MCI.

In sum, we observed two sets of independent and complementary associations with theoretically and clinically relevant phenomena of early cognitive impairment in aging. Both early and more advanced cognitive impairment is likely the result of a complex, life-long series of interactions among various genetic susceptibility and environmental risk factors. Regarding genetic polymorphisms, both *APOE* and *COMT* hold promise for future complex and dynamic analyses of the short- and long-term processes through which some normally aging individuals begin transitioning into preclinical impairment.

### Conflict of interest statement

The authors declare that the research was conducted in the absence of any commercial or financial relationships that could be construed as a potential conflict of interest.
